# Phytochemical screening, antioxidant activity and inhibitory potential of Ficus carica and Olea europaea leaves

**DOI:** 10.6026/97320630015226

**Published:** 2019-03-15

**Authors:** Lahmadi Ayoub, Filali Hassan, Samaki Hamid, Zaid Abdelhamid, Aboudkhil Souad

**Affiliations:** 1 Laboratory of Biochemistry, Environment and Agri-Food (URAC 36) - Faculty of Sciences and Techniques - Mohammedia, Hassan II university Casablanca- Morocco; 2 National Institute of Social Action (INAS), Tangier, Morocco; 3 Environment and Health. Faculty of Sciences, University Moulay Ismail, Meknes Morocco

**Keywords:** Ficus carica L, Olea europaea L, antioxidant activity, phytochemical screening, phenolic, flavonoid contents

## Abstract

It is our interest to screen Oela europaea L and Ficus carica L leaf extract for total phenolic, flavonoid contents and to evaluate their free radical scavenging and Ferric reducing power (FRAP) using 1,1-diphenyl-2-picrylhydrazyl (DPPH). Data shows that Olea europaea and Ficus carica have strong antioxidant potency to scavenge free radical at an optimal phenolic and flavonoid concentration. Results further suggest a strong correlation between antioxidant activities, phenolic and flavonoid contents. Thus, the screening of Ficus carica and Olea europaea leaf extracts for potential antioxidants as source of drugs for several diseases especially oxidative stress and cancers is illustrated.

## Background

The Mediterranean flora is remarkable for its diversity and it is a rich source of medicinal plants [1, 2, 3, 4]. Among them, Ficus carica L. and Olea europaea L. which are widely used in traditional medicine for their benefits as metabolic, respiratory, cardiovascular, antispasmodic, anti-inflammatory, eyesore and cancer diseases remedies [5, 6, 7]. Furthermore previous studies have demonstrated the ability to inhibit the proliferation of several cancer cell lines including pancreatic [8], leukaemia [9], stomach [10], breast [11, 12, 13], prostate [14], carcinoma [15] and colorectal cancer [16]. These pharmacological properties of Ficus carica L and Oela europaea L are probably due to the presence of plant secondary metabolites, which contains several bioactive compounds [17]. Polyphenols [18, 19], flavonoids [19, 20], tannins, organic acids, coumarins, vitamin E and carotenoids have the potency to inhibit the oxidative mechanisms that lead to degenerative diseases [21, 22]. These compounds are able to act as antioxidants by different ways: as reducing agents, hydrogen donators, free radical's scavengers, and singlet oxygen quenchers [23, 24, 25]. This will prevent cell's degeneration. Further knowledge is needed about content of polyphenolics, flavonoids, and antioxidant properties of Ficus carica L. and Oela europaea L. leaves. For this reason we aimed to determine and compare the phytochemical compounds involved, the antioxidant capacity using free radical scavenging activity (DPPH), ferric reducing antioxidant power (FRAP) assays. Moreover, to determine their total phenolics (TPC), total flavonoids (TFC) and investigate their relationship with the antioxidant properties of Ficus carica L and Olea europaea L.

## Methodology

### Plant material and Preparation of Ficus Carica L. and Olea Europaea L. leaves extract:

Ficus carica L. and Olea europaea L. leaves specimens were collected between (July to August for Ficus carica; and September to October for Olea europaea), and dried for twenty day at ambient temperature. Leaves were milled to a fine powder using an electrical mill, and then stored in the dark in closed containers until required. To obtain the plant's extract, 10g of powdered plant was macerated in 250 ml of absolute methanol for 48 h with agitation.

### Phytochemical analysis:

The methanol extract was submitted to phytochemical analysis for secondary metabolites identification using the phytochemical methods, which were previously described [26, 27]. In general, tests for the presence or absence of phytochemical compounds involved the addition of an appropriate chemical agent to the preparation in a test tube. The mixture was then vortexed. The presence or absence of saponins, flavonoids, tannins, and alkaloids were subsequently detected.

### Determination of DPPH free radical scavenging activity:

The ability of Ficus carica L and Olea europaea L extracts to scavenge DPPH free radicals was estimated by the reduction of the color reaction between DPPH solution and sample extracts. For this purpose, we used the method described elsewhere [27]. Briefly, 2 mL of 0.12 mM solution DPPH in methanol was added to 1 mL of various concentrations of each extract (50 - 1000 µg/mL) to be tested. After 30 min at room temperature, the absorbance of the reaction mixture was measured at 517 nm using a spectrophotometer (UNICO, USA). Ascorbic acid (2- 20 µg/mL) was used as positive controls. The scavenger activity was calculated as follows: I% = ((A Control-A Sample)/ A Control) * 100. Where A Control is the absorbance of the blank sample (t = 0 min) and A Sample is the absorbance of the test extract or standard (t = 30 min). The tests were carried out in triplicate. The IC50 values (concentration in µg/mL required to inhibit DPPH radical by 50%) were estimated from the percentage inhibition versus concentration plot, using a Regtox software. The data were presented as mean values ± standard deviation (n = 3).

### Ferric Reducing/Antioxidant Power (FRAP) assay:

The reducing powers of Ficus carica L and Olea europaea L extracts were determined according to the method of [28]. Various concentrations of Ficus carica L and Olea europaea L leaves extracts (50 to 1000 µg per mL) were mixed with 2.5 mL of phosphate buffer (0.2 M, pH 6.6) and 2.5 mL of potassium ferricyanide solution (1%). After incubation in water bath at 50°C for 30 min, 2.5 mL of 10% trichloroacetic acid was added to the mixture to stop the reaction, and the mixture was centrifuged at 3000G for 10 min. The supernatant (2.5 mL) was mixed with 2.5 mL distilled water and 0.1% FeCl3 (0.5 mL) and then the absorbance was measured at 700 nm using a spectrophotometer (UNICO S2100+P, USA). Higher absorbance of the reaction mixture indicates an important reducing power. As positive control, ascorbic acid and trolox were used. All tests were carried out in triplicates to ensure reproducibility.

### Determination of total phenolic compounds content (TPC):

The total phenolic content (TPC) was determined using the Folin- Ciocalteu assay reagent according to the method employed elsewhere [29]. 0.1 mL of extracts (1 mg/mL) was transferred into test tubes and fill up to 4.6 mL with distilled water. After addition of 0.1 mL Folin-Ciocalteu reagent, 0.3 mL of Na2CO3 (2%) solution was added after 3 min. After incubation for 1h30 min at room temperature the absorbance of the mixture was recorded against a blank containing extraction solvent. Gallic acid was used as the standard and TPC in ficus carica L. and olea europaea L. leaves extracts was expressed as milligram of Gallic Acid Equivalents (GAE) per gram of the dry extract averaged from 3 parallel measurements.

### Determination of total flavonoid content (TFC):

Total flavonoid content (TFC) in Ficus carica L and Olea europaea L leaves was determined by colorimetric method used by [30]. Briefly, 0.075 mL of 5% NaNO2 was mixed with 0.5 mL of the sample (1 mg/mL). After 6 min, 0.15 mL of a 10% AlCl3 solution was added and the mixture was putted at ambient temperature for 5 min. Then, 0.5 mL of NaOH (1 M) was added, and the volume was made up to 2.5 mL with distilled water. The absorbance was measured at 510 nm using a spectrophotometer (UNICO, USA), against the blank containing the extraction solvent instead of the sample. The TFC was calculated using a standard calibration of Catechin solution and expressed as micrograms of Catechin Equivalent (CE) per gram of dry extract. All tests were achieved in triplicate.

### Statistical analyses:

The experimental data obtained from the TPC, TFC and antioxidant activity assays were expressed by a mean and standard deviation. To evaluate statistical differences, One-way ANOVA and student's t-test were used. The comparison between the averages was performed through the Duncan test. Correlation coefficient of antioxidant properties was determined by the Pearson test, using GraphPad prism 6. P values = 0.05 were considered statistically significant.

## Results

### Phytochemical screening of Ficus carica L and Oela europaea L leaves:

The results of our preliminary phytochemical analysis of Ficus carica L and Olea europaea L leaves extracts were given in Table 1, which revaled the presence of seven known compounds as: Polyphenols, Alkaloids, Flavonoids, Cumarins, Anthocyanins, Trepenoids and Saponins in Ficus carica L leaves extract. While Olea europaea L leaves extact has shown the presence of all previously tested compounds except the saponins, which have been replaced by Tannins.

### DPPH radical scavenging activity:

**Figure 1:**
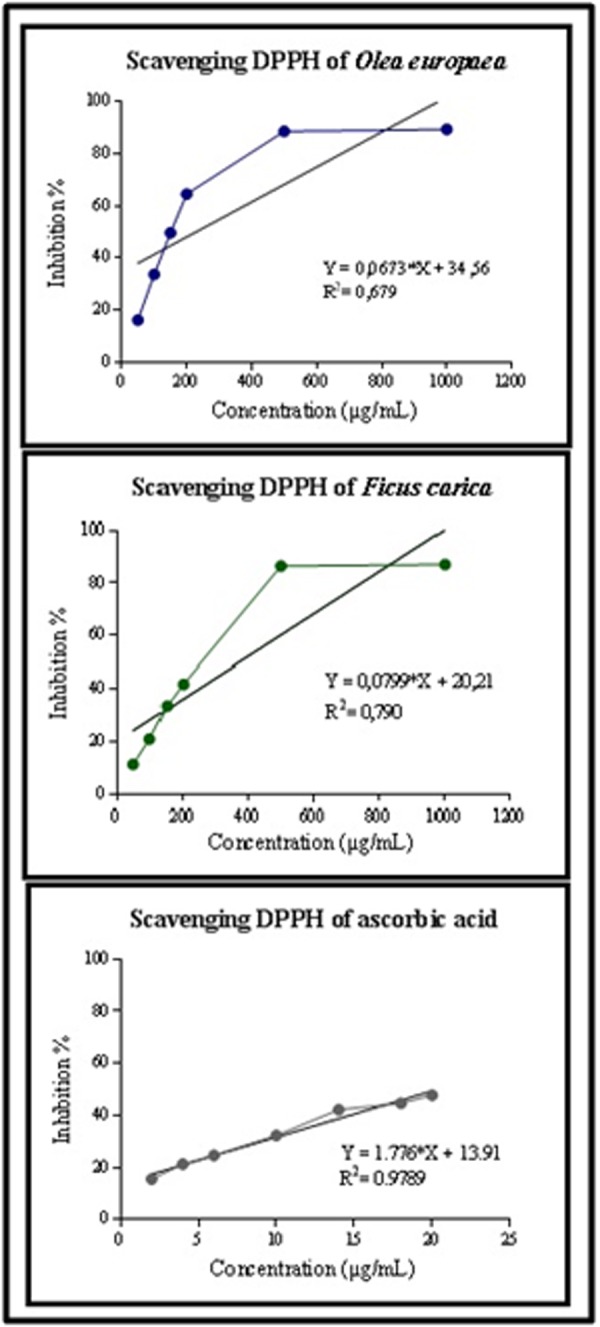
DPPH radical scavenging activity of extracts Ficus carica L, Oela europaea L extracts and ascorbic acid. Data are presented as mean ± SD, n = 3 experiments, p values; *: p < 0.05, **: p < 0.01, ***: p < 0.001.

The abilities of different phenolic compounds from Ficus carica L and Olea europaea L leaves extracts assayed to scavenge to the DPPH+ free radical in comparison to Ascorbic acid under defined conditions was given in Figure 1. The DPPH test of Ficus carica L. showed an increase of the antioxidant activity from (11, 31 ±3, 86%) to (87, 03 ± 0, 15 %) with the increase of extract doses from 50 to 1000 µg/mL. In the same way, the Olea europaea L extratct, showed an important DPPH radical scavenging activity, which was (16, 03 ± 0, 53%) to (89, 44 ±0, 22 %) at the 50 µg/mL to 1000 µg/mL respectively. Furthermore, the results obtained shown an important IC50 of tested extracts of Ficus carica L and Olea europaea L were (275, 23 ±0,045µg/mL; 170,134 ± 0, 06µg/mL, respectively). However, these values were lower than the IC50 measured of ascorbic acid. Our results showed a statistically significant difference between studied extracts (p < 0.05) and positive controls (p < 0.05).

### Reducing power (FRAP) of Ficus carica L and Olea europaea L extracts:

**Figure 2:**
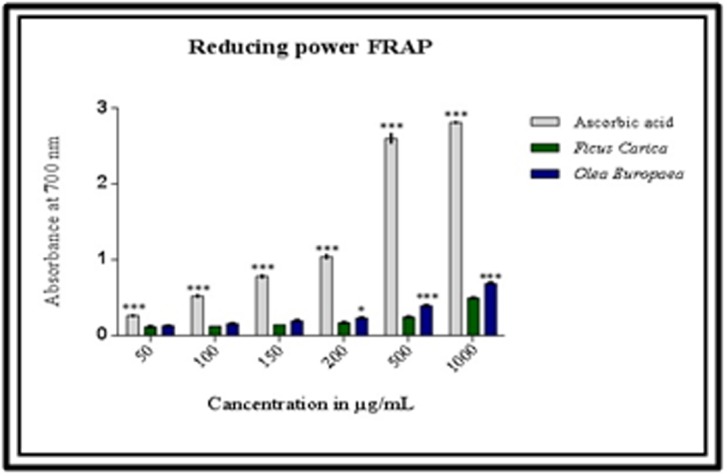
Reducing power of extracts from Olea europaea L, Ficus carica L and ascorbic acid. Data are presented as mean ± SD, n = 3 experiments, p values; *: p < 0.05, **: p < 0.01, ***: p < 0.001.

The reducing power assay (FRAP) of studied plant extracts was investigated and the results are given in Figure 2. The results obtained shown that our extracts had a potency reducing power. In addition, Olea europaea extract showed a higher absorbance which range from 0,125±0,001 to 0,683±0,026 µg/mL than that obtained in Ficus carica extract from 0,113±0,004 to 0,494±0,008. The observed reducing power of both Ficus carica and Olea europaea were dosedependent and increased with increasing amounts of extracts. However, the reducing power of ascorbic acid varied from 0,260± 0,014 to 2, 81± 0,014. This difference was statistically significant.

### Total phenolics (TPC) and flavonoids (TFC) contents measurement:

The concentration of total phenolics (TPC) was determined using spectrophotometric analysis with Folin Ciocalteau's phenolic reagent as shown elsewhere [30]. The determined TPC value was given as Equivalent Gallic Acid using an equation obtained from a standard gallic acid graph (R2 = 0.992). As shown in Table 2, the concentration of TPC both in Ficus carica L and Olea europaea L leaves extracts are (96,46 ± 0, 42 µg GAE/mg of dry extract) and (125, 92 ± 0, 68 µg GAE / mg of dry extract) respectively. Theses measured concentration of tested plants is higher than that of control test (0, 57 ± 0, 14 µg GAE/mg of dry extract). On the other hand, the total flavonoids contents (TFC) was determined by using a calibration curve of Catechin (R2=0,988). The results obtained suggests an important concentration of TFC in Ficus Carica L and Olea Europaea L leaves extract with an average of (33, 52 ± 1, 34 µgCE/mg of dry extract ; 22, 18 ± 2, 89 µgCE/mg of dry extract) respectively. The results are statistically significant in comparison with the control (1, 18 ± 0, 04 µgCE/mg of dry extract).

### Correlation between antioxidant activities, phenolic contents and flavonoid contents:

The evaluation of the correlation between antioxidant activity, phenolic and flavonoid contents are given in Table 3. The results obtained demonstrate an important correlation between antioxidant activity, phenolic and flavonoids compounds both for Ficus carica and Olea europaea. Furthermore, all R2 values were statistically significative at p<0.01. the DPPH values were strongly correlated with FRAP assays both in Ficus carica and Olea europaea with a R2=0.957 and R2=0.968 respectively. In addition, the antioxidant results obtained from DPPH were strongly correlated with the total phenolic contents (R2=0.953 for Ficus carica ; R2=0.973 for Olea europaea) and total flavonoid contents (R2=0.922 for Ficus carica ; R2=0.934 for Olea europaea).

## Discussion

The oxidative stress has been implicated in numerous diseases like atherosclerosis [31], cardiovascular diseases [32, 33], aging [34], diabetes [35], neurodegenerative diseases and cancer [36, 37, 38, 39]. To avoid this problem, scientific researchers have returned to folk medicine to investigate and find certain bioactive molecules, which may offer resistance against oxidative stress by scavenging free radicals and inhibiting lipid peroxidation [40]. In this study, we aim to determine the phytochemical compounds of two Moroccan plants extract namely Ficus carica and Olea europaea, to investigate their antioxidant properties using DPPH and FRAP methods. Furthermore, we are considering establishing the correlation between their antioxidant activities and the flavonoid and total phenolic contents. The qualitative phytochemical analyses of these extracts showed the presence of major known family compounds like polyphenols, alkaloids, flavonoids, cumarins, anthocyanins, trepenoids, saponins and tannis (Table 1). Some screening compounds of our preliminary phytochemical analyses have been reported previously [26].

## Antioxidant activity of Ficus carica and Olea europaea:

**Figure 3:**
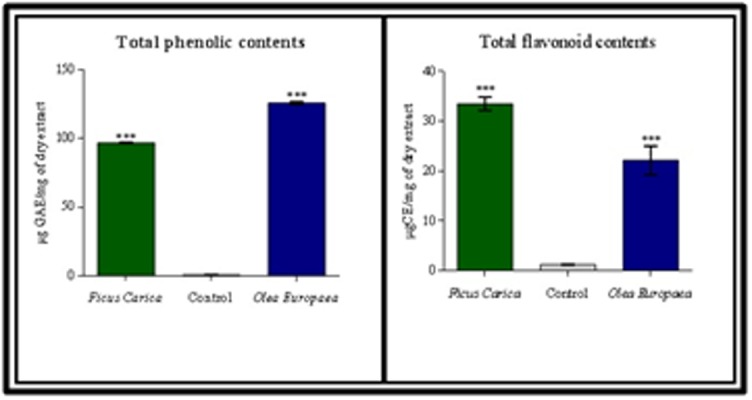
A) Total polyphenol content expressed as gallic acid equivalents (µg GAE)/mg plant extract; B) Total flavonoid content expressed as quercetin equivalents (µg QE)/mg plant extract. Data are presented as mean ± SD, n = 3 experiments, p values; *: p < 0.05, **: p < 0.01, ***: p < 0.001.

We have using tow known's methods for this purpose, the first method was the Free Radical Scavenging (DPPH), which is stable at room temperature with a violet colorization, in the presence of an antioxidant molecule it reduced and giving rise to uncolored solution [27]. The second method was the Ferric Reducing Power (FRAP), which based on reduction of ferric ions (Fe3+)-ferricyanide complexes to ferrous (Fe2+) form by an antioxidant in acidic pH [28]. The results of DPPH assays suggested that the tested plants extracts possessed a strong antioxidant activity which vary from (11, 31 ±3, 86%) to (87, 03 ± 0, 15 %) and from (16, 03 ± 0, 53%) to (89, 44 ± 0, 22 %) for Ficus carica and Olea europaea respectively (Figure 1). Furthermore, this activity increase progressively by increasing the concentration of extracts, this observed activity was dose-dependent. The obtained results are in concordance with others reported previously [41, 42]. In addition, the FRAP assays of our extracts have demonstrated an antioxidant potency, which was also dose-dependent, the observed results were in agreement with previously found [41, 42]. The found results could be explain the important ability of our extracts to scavenging free radical such as ROS, inhibiting lipid peroxidation, avoiding DNA damage and prevent carcinogenesis processes [22]. This strong antioxidant activity of Ficus carica and Olea europaea leaves may be due to the affluence of secondary metabolites such as alkaloides, flavonoids and polyphenols. Which prompted us to study and determine total phenolic and flavonoid contents (Figure 3), the found results confirmed our hypothesis and suggest that both Ficus carica and Olea europaea leaves have an important concentration of phenolics and flavonoid compounds (Table 2), our results were in agreement with numerous founded both in Ficus carica and Olea europaea [18, 19]. The present work suggests a strong correlation between antioxidant activities and a high content of phenols (Table 3), these results was concordant to other reported study [44], which means that phenols compounds are the main agents responsible and contribute largely in the antioxidant activities of medicinal plants [43, 44]. Moreover, the anti-radical ability of phenolic compounds is due to their capacity to trap free radicals through the transfer of the hydrogen atom then transformed into a stable molecule [45], and their reducing power is due to the presence of hydroxyl group in their structure that can serve as an electron donor [46].

## Conclusion

The aim of this study was to test whether Ficus carica L and Olea Europaea L leaves used for traditional medicine practices could be promising sources of natural antioxidants. The robust linear correlations observed between phenolic, flavonoid and antioxidant capacity determined by the DPPH assay and FRAP assay suggest that phenolic and flavonoid contents could be used as an indicators of antioxidant properties. The knowledge of traditional medicine practices can be a source of useful information for the isolation of natural extracts to develop new products for natural health care and well-being of domestic animals. Further investigations for potential applications of new natural antioxidants require anyway, elucidation of the chemical composition of phenolic and flavonoid in vivo studies in order to better establish the functionality of the examined plant species.

## Conflict of Interest

Authors declare no conflict of interest

## Figures and Tables

**Table 1:** Phytochemical constituents of Ficus carica L and Olea europaea L leaves

	Ficus carica L.	Olea europaea L.
Polyphenols	+	+
Alkaloids	+	+
Tannins	-	+
Flavonoids	+	+
Saponins	+	-
Cumarins	+	+
Anthocyanins	+	+
Anthraquinons	-	-
Trepenoids	+	+

**Table 2:** Flavonoid and phenolic content

Species	TPC (µg GAE/mg of dry extract)	TFC(µg CE/mg of dry extract)
Ficus Carica L.	96,46 ± 0,42	33,52 ± 1,34
Olea Europaea L.	125,92 ± 0,98	22,18 ± 2,89
Control	0,57 ± 0,14	1,18 ± 0,04

**Table 3:** Correlation coefficient among antioxidant assays, total phenolic contents and total flavonoid contents

Ficus carica	DPPH	FRAP	TPC	TFC
DPPH	_	0.957	0,953	0.922
FRAP	0.957	_	0.936	0.93
TPC	0.953	0.936	_	0.965
Olea europaea	DPPH	FRAP	TPC	TFC
DPPH	_	0.968	0,973	0.934
FRAP	0.968	_	0.951	0.94
TPC	0.973	0.951	_	0.982

## References

[1] Djeridane A (2006). Food Chem.

[2] Bremness L (2001). Plantes aromatiques et medicinales.

[3] Gonzalez Tejero MR (2008). Journal of Ethno pharmacology.

[4] BULLITTA S (2007). Genetic Resources and Crop Evolution.

[5] Duke JA (2002). Hand Book of Medicinal Herbs CRC PressBoca Raton Fla USA.

[6] Werbach M (1993). Healing with Food Harper Collins New YorkUSA.

[7] Guarrera PM (2005). Fitoterapia.

[8] Goldsmith CD (2015). Molecules.

[9] Samet I (2014). Oxid Med Cell Longev.

[10] Hashemi SA (2011). Iranian Red Crescent Medical Journal.

[11] Barrajon Catalan E (2015). J Pharm Biomed Anal.

[12] Quirantes Pine R (2013). J Pharm Biomed Anal.

[13] Elamin MH (2013). Food Chem Toxicol.

[14] Acquaviva R (2012). Int J Oncol.

[15] Teixeira DM (2006). J Chromatogr A.

[16] Cardeno A (2013). Cancer.

[17] Pereira AP (2007). Necessidades humanas: subsidios a critica dos m�nimos Editora Cortez.

[18]  Verberic R (2007). Food chemistry.

[19] Vlahov G (1992). Journal of the science of food and agriculture.

[20] Du Toit R (2001). Toxicology.

[21] Silva BM (2004). J Agric Food Chem.

[22] Soobrattee MA (2005). Mutation Research/Fundamental and Molecular Mechanisms of Mutagenesis.

[23] Merken HM, Beecher GR (2000). J Agric Food Chem.

[24] Costa RM (2009). Food Chem.

[25] Fattouch S (2007). J Agric Food Chem.

[26] Dohou N (2003). Bull SocietePharm Bordx.

[27] BOIS MS (1958). Nature.

[28] OYAIZU M (1986). Jpn J Nutr.

[29] Tsai TH (2008). Food Chem.

[30] Kim DO (2003). J Agric Food Chem.

[31] Singh U, Jialal I (2006). Pathophysiology.

[32] Csanyi G, Miller Jr (2014). FJ Int J Mol Sci.

[33] Vijaya Lakshmi SV (2013). Indian Journal of Biochemistry and Biophysics.

[34] Romano AD (2010). Journal of Nephrology.

[35]  aynes JW (1991). Diabetes.

[36] Chen X (2012). Neural Regen Resv.

[37] Poli G (2004). Curr Med Chem.

[38] Klaunig JE, Kamendulis LM (2004). Ann Rev PharmacolToxicol.

[39] Burcham PC (1998). Mutagenesis.

[40] Vivek KB (2014). Asian Pacific Journal of Tropical Medicine.

[41] Silva S (2006). Food Science and Technology International.

[42] Dekdouk N (2015). Evidence-Based Complementary and Alternative Medicine.

[43] Ramos-Escudero F (2013). International Journal of Food Properties.

[44] Piluzza G, Bullitta S (2011). Pharmaceutical Biology.

[45] Huang WY, Cai YZ (2010). Nutr Cancer.

[46] Siddhuraju P, Becker K (2007). Food Chem.

